# A reproducible workflow for assembling the mitochondrial genome of *Acheta domesticus* (Orthoptera: Gryllidae)

**DOI:** 10.1002/ece3.11696

**Published:** 2024-07-04

**Authors:** Somjit Homchan, Wibhu Kutanan, Yash Munnalal Gupta

**Affiliations:** ^1^ Department of Biology, Faculty of Science Naresuan University Phitsanulok Thailand; ^2^ Center of Excellence for Innovation and Technology for Detection and Advanced Materials (ITDAM) Naresuan University Phitsanulok Thailand

**Keywords:** galaxy workflow, house cricket, insects, mitochondrial genome, phylogeny

## Abstract

In this study, we report the assembly and annotation of the mitochondrial genome (mitogenome) of *Acheta domesticus* from breeding facility, a species commonly known as the house cricket. This species is considered to be an important edible cricket. The mitogenome was assembled using a reproducible protocol implemented on the Galaxy Europe Server, which involved uploading paired‐end fastq reads for bioinformatic analysis. The resulting mitogenome is 15,784 base pairs in length and has a GC content of 29.05%. The nucleotide composition of this mitogenome is similar to that of other insect mitogenomes, with A, T, C, and G nucleotides comprising 39.2%, 31.7%, 19.6%, and 9.5% of the mitogenome, respectively. The gene organization of the *A. domesticus* mitogenome is identical to that of other cricket species. The mitogenome consists of 37 genes, including 13 protein‐coding genes, 22 tRNA genes, and two rRNA genes. The congruence between PCA and Bayesian evolutionary tree analysis in clustering the divergent *A. domesticus* sequences highlights these genomes as candidates for further study to elucidate their distinct features and evolutionary history.

## Graphical abstract

This study presents the comprehensive assembly and analysis of the complete mitochondrial genome of the economically important house cricket, *Acheta domesticus*. The high‐quality mitogenomic data obtained through our robust bioinformatic workflow serve as an invaluable resource for precise DNA barcoding, species identification, and evolutionary studies within the order Orthoptera. The comparative genomic analysis and phylogenetic insights derived from this research shed light on the evolutionary relationships and genetic diversity of *A. domesticus* and related cricket species.

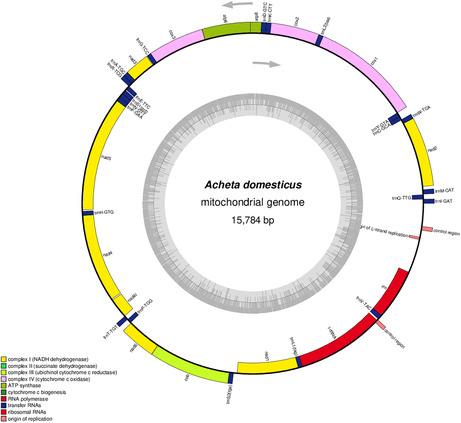

## 1 INTRODUCTION

Entomophagy, the practice of consuming insects, is gaining popularity worldwide, although it has long been practiced in certain Asian and African, particularly in rural communities (Gahukar, 2020; van Huis, 2018). In Thailand, house crickets (*Acheta domesticus*) are highly sought after by locals due to their distinctive flavor and crispy texture (Gupta et al., 2020). Given the inexpensive cost of rearing and maintaining house crickets, even local residents are increasingly involved in breeding and selling these insects in local markets (Hanboonsong et al., 2013). As the trend of insect consumption grows, there is a clear need for reliable and rapid insect identification techniques that can be combined with next‐generation sequencing (NGS) data and bioinformatic analysis.

The phylum Arthropoda comprises the majority of animal biodiversity, constituting approximately 80% of all known species in the animal kingdom, with a documented total of 1.5 million species (Stork et al., 2015). Insects are also increasingly being used in the production of commercial goods. Thus, scientific identification is crucial before using insects for commercial purposes, as entomophagy continues to rise across the globe, necessitating the development of databases and strategies for food security (Wolf‐Hall & Nganje, 2017). In terms of food security, scientific identification is required before using them to produce commercial item. Insects present unique challenges for entomologists in terms of identification, not only due to their size but also because of the vast number of species and the subtle morphological differences between closely related species. These factors make accurate species identification more dependent on genetic tools than on traditional morphological methods alone. Consequently, molecular markers are an efficient solution for species identification and have also been employed in the highly specialized field of forensic sciences (Loxdale & Lushai, 1998). However, several factors must be addressed while selecting a molecular marker. The chosen fragment must possess characteristics that facilitate isolation and amplification, and exhibit a mutation rate that is sufficient for distinguishing among different species, yet slow enough to minimize intraspecific genetic variation (Patwardhan et al., 2014; Spooner, 2005).

The mitochondrial genome is a compact, circular DNA molecule found within the mitochondria of eukaryotic cells. It plays a vital role in energy metabolism and cellular functioning (Gustafson et al., 2020). The study of mitochondrial genomes has attracted increased attention in the fields of genetics and evolutionary biology in recent years. Mitochondrial DNA (mtDNA) changes at a quicker pace compared to DNA packed in cell nucleus (Geetha et al., 2011), allowing for a more exact determination of evolutionary relationships among closely related species. Therefore, of the mitochondrial cytochrome c oxidase subunit I (COI) is commonly used for DNA barcoding for unknown species identification (Roe & Sperling, 2007). However, finer classification can be achieved by employing whole mitochondrial genome to establish evolutionary relationship among related species. Moreover, lack of intermolecular recombination, maternal inheritance, rapid evolutionary rate, and large copy number of mtDNA, it has been widely used for the analysis of metazoan phylogenetic relationships at various taxonomic levels (Gu et al., 2007; Janke et al., 1997). The mtDNA is a great resource for studying organism evolution. Researchers can infer links between insect species and construct phylogenetic trees by comparing mitochondrial genomes (De Mandal et al., 2014). Recently, researchers employed both long‐ and short‐read sequencing techniques to demonstrate the dynamic evolution of mitochondrial genome sizes in beetles (Morgan et al., 2022). Furthermore, the mitochondrial genome can be used to distinguish between species and detect hybridization occurrences. Previous study has also found that the mitochondrial genome accumulates a significant number of neutral mutations (Holyoake et al., 2001), making it easier to identify species based on alteration in mtDNA. mtDNA regions (COI gene, COII gene, and 12s rRNA gene) are frequently utilized for species identification and discrimination (Patwardhan et al., 2014), but combining entire mtDNA genome sequence with bioinformatics gives a valid method for categorizing insects for future food security. Furthermore, the use of mitochondrial genome data in the automation of insect species identification can be significantly enhanced by incorporating machine learning techniques. Researchers can train machine learning algorithms to identify species or predict specific features by utilizing the mitochondrial genome sequence as a feature (Bhadola & Gupta, 2022).

The full genome data of *A. domesticus* are being used in this project to assemble a complete mitochondrion. Therefore, the raw Fastq files (forward and reverse reads) were used to extract mitochondrial DNA reads from the whole genome sequencing data using mitochondrial genome assemblers such as NOVOPlasty (Dierckxsens et al., 2017). The mitochondrial genome of *A. domesticus* will aid in understanding evolutionary history of this important edible cricket species. Additionally, the techniques utilized in this study can enable the reconstruction of the mitochondrial genome from extensive genomic sequencing data.

## 2 MATERIALS AND METHODS

### 2.1 Sequence assembly

We previously sequenced the genome of *A. domesticus* in order to develop microsatellite markers. The assembled genome was deposited in the NCBI GenBank under accession number GCA_014858955.1. For this study, the NGS reads were utilized for the assembly of the mitochondrial genome. A reproducible protocol was created on the Galaxy Europe Server by uploading paired‐end fastq reads for bioinformatic analysis (Jalili et al., 2020). Assembly pipeline is accessible for download and execution through the following Link: https://usegalaxy.eu/u/yashm/w/mitochondrial‐genome‐assembly‐v2.

The paired‐end fastq reads were first checked for quality using FastQC (Brown et al., 2017), and then trimmed using the Trimmomatic tool (Bolger et al., 2014). The trimmed forward and reverse reads were used for de novo assembly with NOVOPlasty (Dierckxsens et al., 2017). The final assembly was annotated using MITOS (Bernt et al., 2013), and then aligned to nucleotide sequences using BLAST (Altschul et al., 1990) for secondary verification of annotated CDS features. tRNAscan (Chan & Lowe, 2019) was utilized to confirm the tRNA annotations found in the mitochondrion. Additionally, 22 tRNA genes were manually inspected to check for the presence of anticodons for respective amino acids. The annotated mitochondrion of *A. domesticus* was submitted to NCBI GenBank (Benson et al., 2012) under accession number OK504623.1. The mitochondrial genome map was visualized utilizing the OrganellarGenomeDRAW (OGDRAW) version 1.3.1 server (Greiner et al., 2019) utilizing the GenBank file generated by the National Center for Biotechnology Information (NCBI).

### 2.2 Phylogenetic analysis

In this study, phylogenetic analysis was conducted using complete mitochondrial genomes of 17 species, including the newly assembled mitochondrial genome of *A. domesticus*. To begin the analysis, 17 mitochondrial sequences were obtained from GenBank using NCBI BLAST, with the mitochondrial genome of *A. domesticus* as the query sequence. These sequences were then aligned using MUSCLE (Edgar, 2004) and the best model for phylogenetic analysis was determined using jModelTest 2.1.10 (Darriba et al., 2012). The general time reversible model with gamma distributed with invariant sites (GTR + G + I) was chosen as it had the lowest BIC score (127417.92383). The phylogenetic tree was constructed using Bayesian Inference with BEAST Version 2.6.6, using Markov chain Monte Carlo for 100 million generations with the following substitution model rates: R(a) [AC] = 3.0125, R(b) [AG] = 7.5363, R(c) [AT] = 3.1960, R(d) [CG] = 2.6453, R(e) [CT] = 29.6462, and R(f) [GT] = 1.0000 (Drummond et al., 2012). The tree was then visualized graphically using FigTree Version 1.4.4 (Rambaut, 2009).

### 2.3 PCA analysis

Study utilized principal component analysis (PCA) as a means to investigate the underlying structure of mitochondrial DNA sequences across complete mitochondrial genomes of 17 species. The DNA sequences were employed for an initial standardization process wherein any nucleotide characters that deviated from the standard were substituted with the symbol “N.” The standardized sequences were subsequently encoded numerically using a label encoder, wherein the nucleotides A, C, G, T, and N were translated into corresponding numerical values. In order to accommodate sequences of different lengths, the technique of zero‐padding was employed to align all sequences to a uniform length. Subsequently, the padded and encoded sequences were employed for PCA utilizing the scikit‐learn library in the Python programming language. For visualization purposes, the first two principal components were extracted to represent the primary directions of variability within the sequences. The application of KMeans clustering was utilized to classify sequences into discrete clusters. The identification of outliers was conducted by measuring their Euclidean distance from the centroid of the plot. This distance was computed using the interquartile range (IQR) as a threshold. We are delighted to inform you about the release of our Python script specifically developed for conducting PCA on mitochondrial DNA sequences. The script was devised as a component of a research endeavor aimed at examining the fundamental organization of entire mitochondrial genomes. The script is available for access on our GitHub repository (https://github.com/yashmgupta/PCA‐analysis_mtgenomes).

## 3 RESULTS

The main focus of this study was to assemble the complete mitochondrial genome of *A. domesticus* for DNA barcoding purposes. A protocol was developed on the Galaxy Europe server (Jalili et al., 2020) that used raw sequence data for the assembly process. The final assembly consisted of 789,352 reads and resulted in a 15,784 base pair circularized sequence with a GC content of 29.05%. The mitochondrial DNA had an average coverage of 8309x. This information can be utilized in DNA barcoding and understanding the genetic makeup of *A. domesticus*.

**FIGURE 1:**
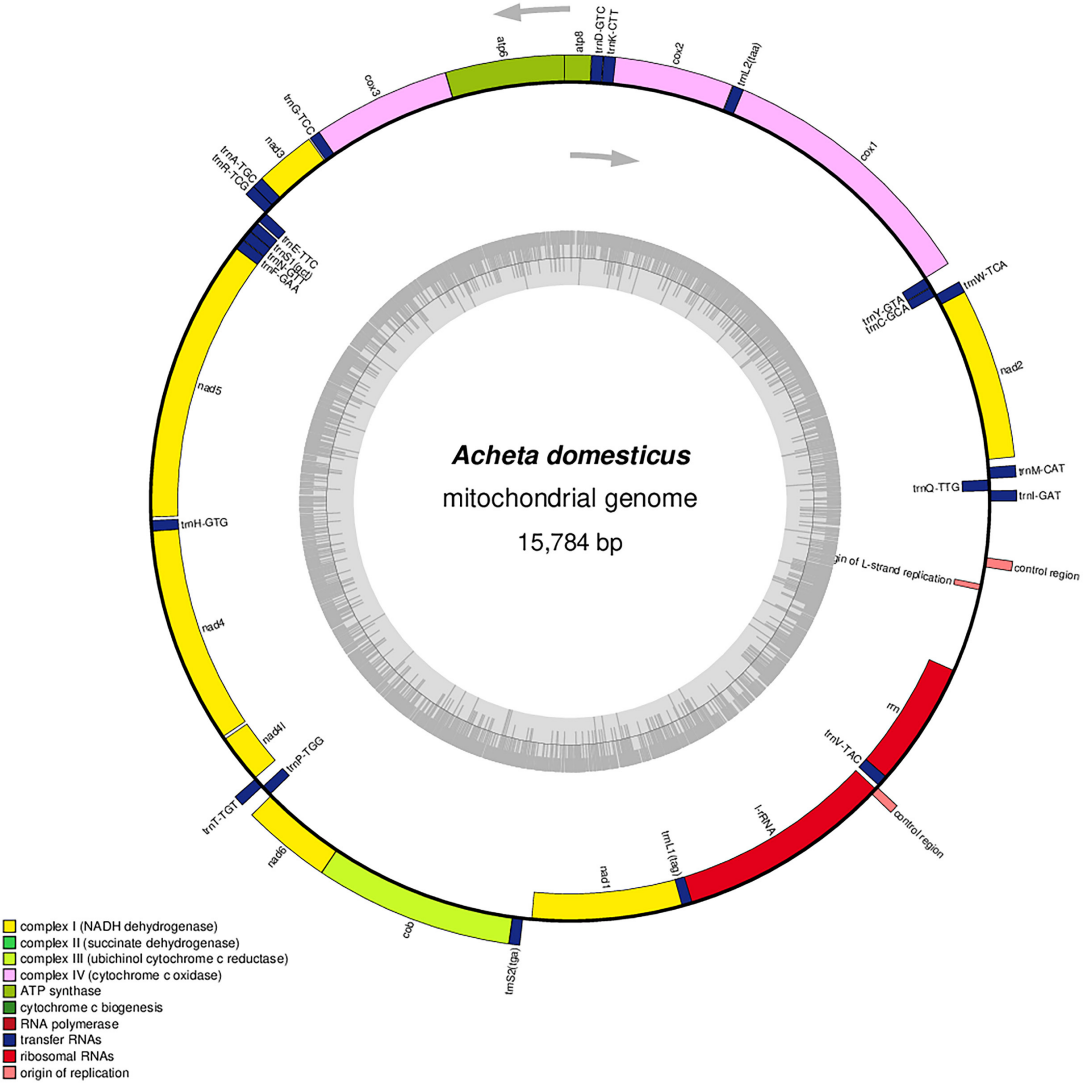
The mitochondrial genome map of *Acheta domesticus* shows the arrangement of all 37 genes, with the GC% along the mitochondrion represented by the inner circle.

The nucleotide composition of mitochondrial genome of *A. domesticus* (Accession number: OK504623.1) was consistent with other insect mitochondrial genomes, with 39.2% A, 31.7% T, 19.6% C, and 9.5% G. Additionally, the organization of genes in the mitogenome of *A. domesticus* was found to be identical to that of other cricket species. The final assembly of the mitochondrial genome of *A. domesticus* was revealed to contain 37 genes, which include 13 protein‐coding genes, 22 transfer RNAs (tRNAs), and 2 ribosomal RNAs (rRNAs). The order of these genes is as follows: Ile, Gln, Met, NAD2, Trp, Cys, Tyr, COX1, Leu‐2, COX2, Lys, Asp, ATP8, ATP6, COX3, Gly, NAD3, Ala, Arg, Glu, Ser‐1, Asn, Phe, NAD5, NAD4, His, NAD4L, Thr, Pro, NAD6, COB, Ser‐2, NAD1, Leu‐1, L‐rRNA, Val, S‐rRNA. A graphical representation of the annotated mitochondrial genome map of *A. domesticus* is presented in Figure 1.

**FIGURE 2:**
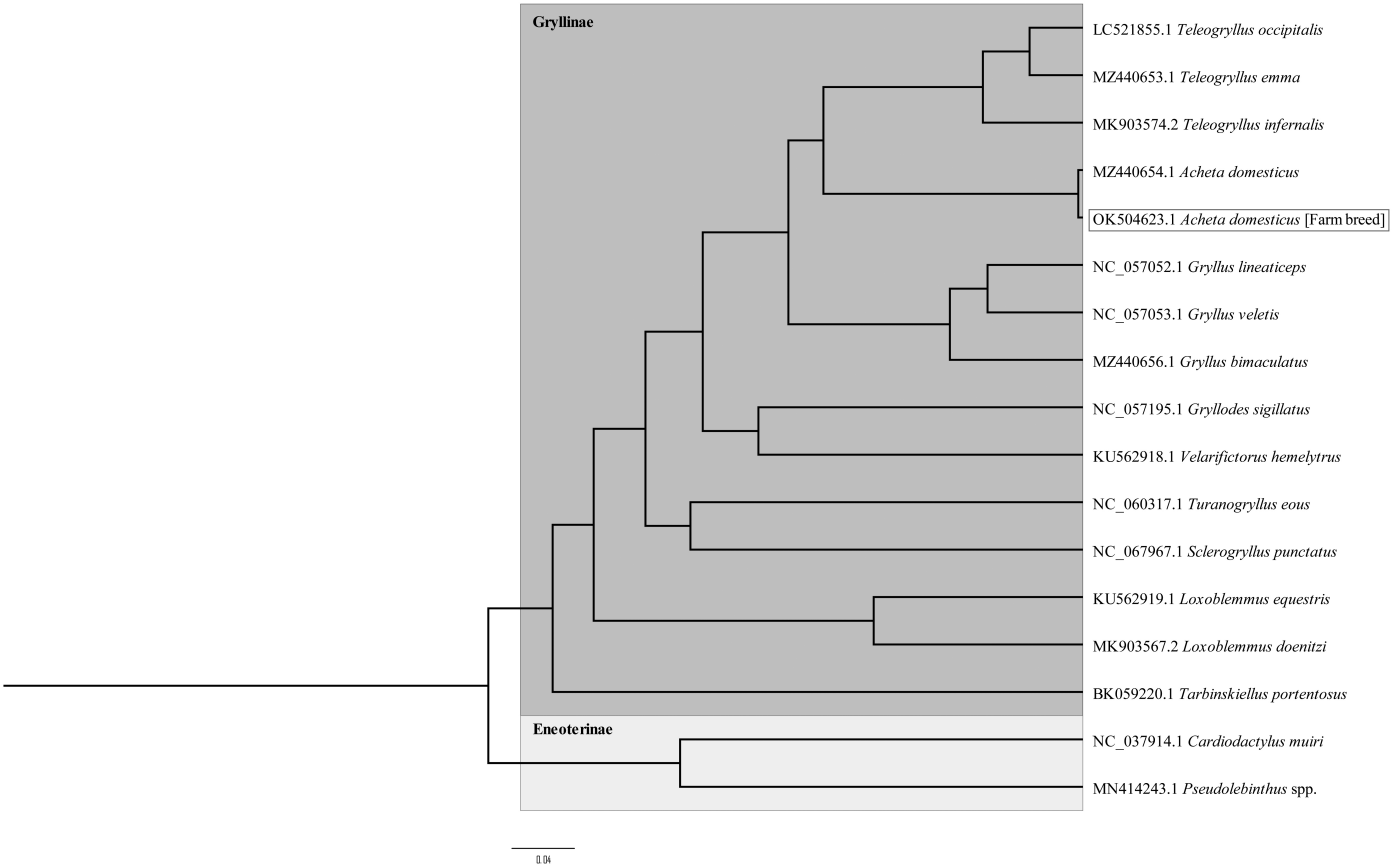
The evolutionary history was inferred for maximum clade credibility tree using a Bayesian inference approach and the General Time Reversible model, with 17 mitochondrial sequences analyzed. *Cardiodactylus muiri* and *Pseudolebinthus* spp. were utilized as outgroup taxa to provide a comparative analysis.

In order to uncover the evolutionary relationship of *A. domesticus* with other related cricket species, a phylogenetic analysis was conducted using the complete mitochondrial DNA sequences. The results, depicted in Figure 2, revealed significant insights into the evolutionary positioning of *A. domesticus*. The analysis indicates that *A. domesticus* belongs to the monophyletic family Gryllinae. Within this family, *A. domesticus* forms a distinct clade with species from the genera *Teleogryllus* and *Gryllus*. Specifically, *A. domesticus* (both the wild type and the farm‐bred strain) groups closely with *Teleogryllus emma* and *Teleogryllus infernalis*, suggesting a close evolutionary relationship among these species. This finding implies that *A. domesticus* shares a significant portion of its evolutionary history with these *Teleogryllus* species, underscoring its phylogenetic affinity within the Gryllinae family. Moreover, the inclusion of various *Gryllus* species such as *Gryllus lineaticeps*, *Gryllus veletis*, and *Gryllus bimaculatus* further delineates the evolutionary relationships within the Gryllinae. The presence of other species like *Gryllodes sigillatus*, *Velarifictorus hemelytrus*, and *Turanogryllus eous* within the same family clade provides a comprehensive view of the genetic diversity and evolutionary pathways among these crickets. Additionally, the analysis revealed that the two species from the Eneopterinae outgroup, *Cardiodactylus muiri* and *Pseudolebinthus* spp., are situated within a separate clade from the Gryllinae. This separation highlights the distinct evolutionary lineage of the Eneopterinae in contrast to the Gryllinae.

**FIGURE 3:**
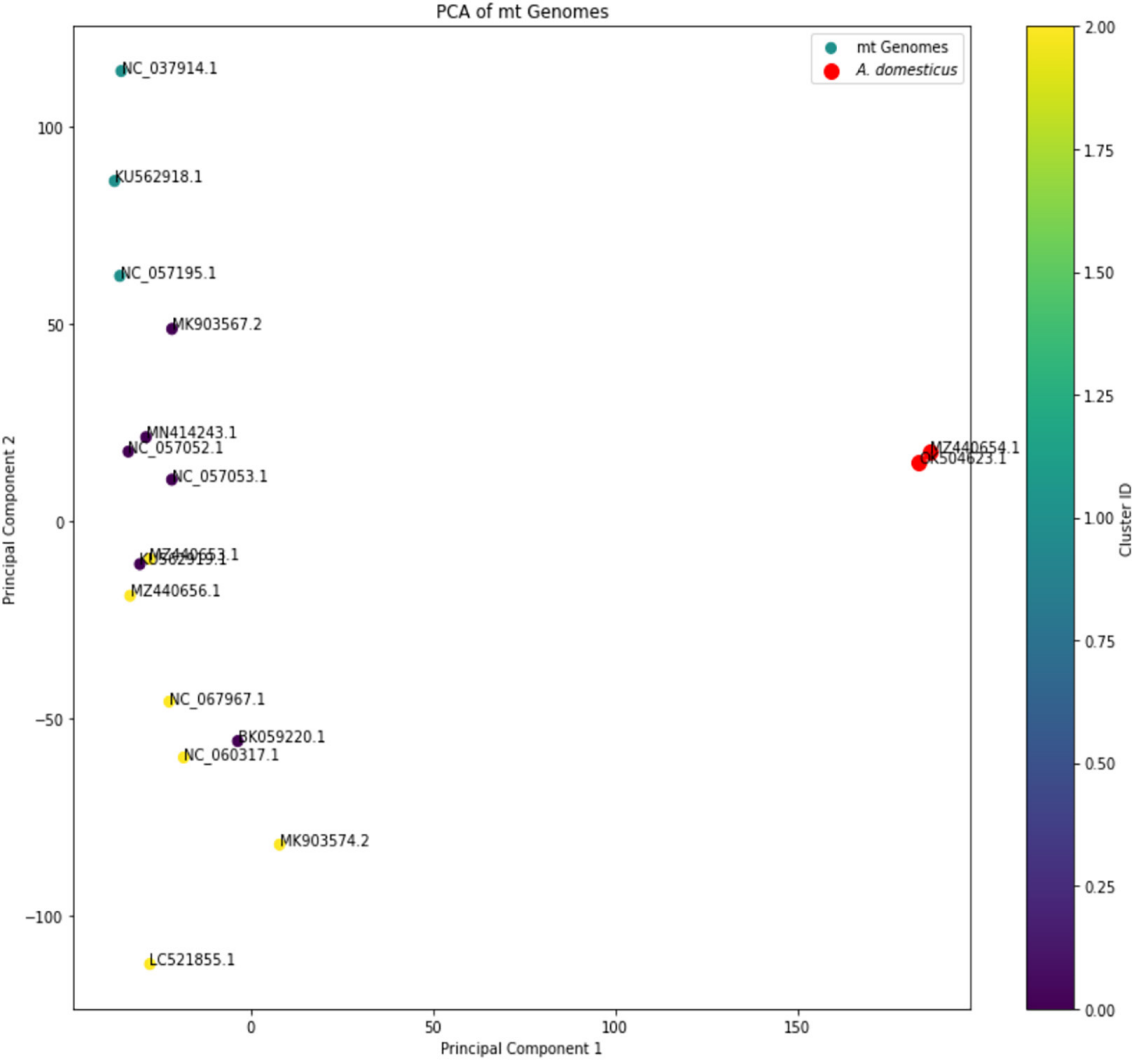
The PCA plot for mt genomes is displayed above. Each point represents a sequence, and the color indicates the cluster to which it belongs, as determined by K‐means clustering. Sequence IDs are annotated directly on the scattered plot. *Acheta domesticus* sequences cluster together, indicating their high similarity. However, their sequences are distinct enough to set them apart in the PCA plot.

PCA plot provides a reduced‐dimensionality view of the sequence data, capturing the major variations among the 17 mitochondrial genomes (Figure 3). The sequences generally cluster together, indicating a high level of similarity among most of them. However, outliers were identified in red, specifically “OK504623.1 *A. domesticus* breed Farm mitochondrion, complete genome” and “MZ440654.1 *A. domesticus* mitochondrion, complete genome.” Intriguingly, both outliers belong to the same species, *A. domesticus*, but their sequences are distinct enough to set them apart in the PCA plot.

## 4 DISCUSSION

**FIGURE 4:**
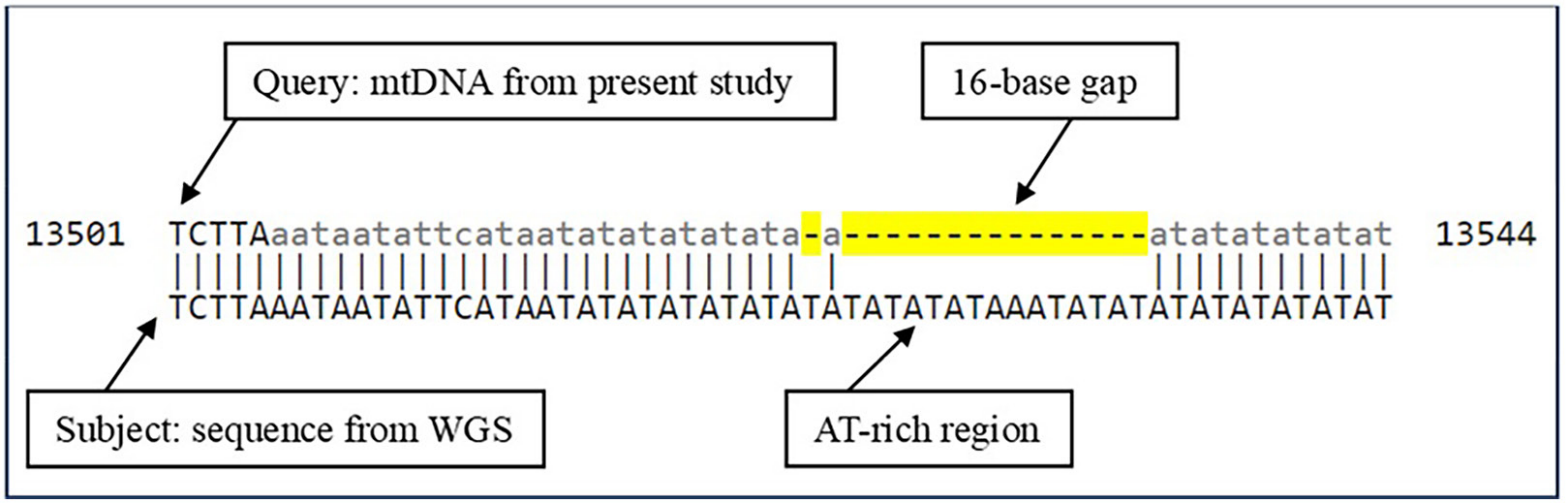
BLAST alignment shows comparison between an assembled mitochondrial genome sequence and sequence from previously published whole genome sequencing (WGS) data. The alignment highlights an indel (insertion/deletion) variation within a repetitive AT‐rich region, where the mitochondrial genome sequence has a 16‐base gap compared to the anchored sequence from the published data. This gap is represented by the dashed line in the middle of the alignment.

The complete mitochondrial genome sequence of *A. domesticus* presented in this study provides a valuable genetic resource for DNA barcoding and investigating the evolutionary relationships of this important insect species. Consistent with other insect mitochondrial genomes, the 15,784 bp circular genome exhibits a typical nucleotide composition and gene organization, harboring 37 genes including 13 protein‐coding genes, 22 tRNAs, and 2 rRNAs. The high coverage depth of 8309x ensures the accuracy and reliability of the assembled mitochondrial genome sequence. To further validate our approach, we have utilized the assembled mitochondrial genome in this study to compare with a previously published assembled whole genome (Dossey et al., 2023), anchored sequence demonstrating 99.88% identity with 100% query coverage. The key difference lies in a stretch of adenine (A) and thymine (T) repeats, likely due to insertion/deletion variations within this repetitive region. Figure 4 displays a BLAST alignment between the assembled mitochondrial genome and anchored sequence from previously published whole genome data. The alignment highlights an indel variation within a repetitive AT‐rich region, where the mitochondrial genome has a 16‐base gap compared to the anchored sequence. This mitogenomic data not only enables precise species identification through DNA barcoding but also lays the foundation for comparative genomic studies and phylogenetic analyses within the order Orthoptera.

**FIGURE 5:**
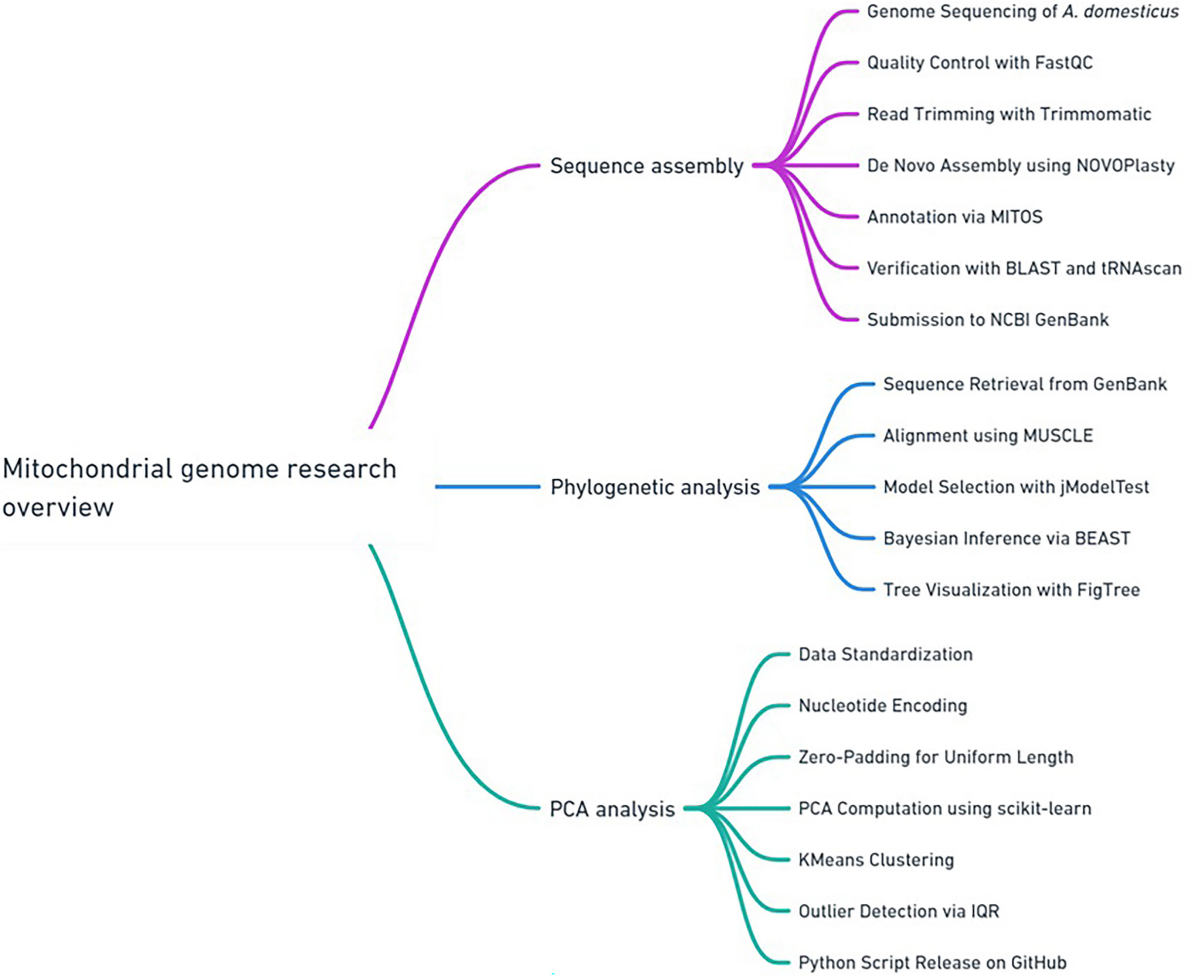
Comprehensive workflow of mitochondrial genome research, covering sequence assembly, phylogenetic analysis, and PCA analysis.

The assembled mitochondrial genome of *A. domesticus* provides a high‐quality genetic resource that can be applied to other mitochondrial genome projects. This resource is particularly valuable for studying population genetics and evolutionary biology within Orthoptera. This study highlights the effectiveness of NGS methods in achieving comprehensive and accurate mitochondrial genome assemblies. The high coverage and reliability of the assembled genome ensure its utility for further genomic and evolutionary studies. To enhance comprehension and facilitate execution, the complete workflow is visually illustrated in Figure 5. Older methods of constructing mitochondrial genomes, such as PCR‐based approaches, have significant limitations, including error‐proneness and inefficiencies (Kinkar et al., 2021; Legati et al., 2021). In contrast, NGS offers high coverage and precision, making it the preferred approach for mitochondrial genome assembly. This study demonstrates that NGS, combined with advanced bioinformatic tools, can overcome the challenges of sequencing complex regions, providing a detailed and accurate mitochondrial genome (Al‐Nakeeb et al., 2017; Hu et al., 2021; Song et al., 2022).

In addition to the challenges mentioned above, the generation of mitochondrial genome assemblies can also be hindered by the production of incorrect assemblies. These assemblies, which are often labeled as UNVERIFIED sequences on GenBank (such as MK204368.1), can be the result of a variety of factors, including errors in sample preparation, sequencing, or bioinformatics. This can lead to inaccuracies in the final sequence and can hinder downstream analysis. It is important for researchers to carefully evaluate and verify the accuracy of their assemblies before submitting them to GenBank or using them for further analysis.

In a previous study, we employed a toolset to generate a mitochondrial genome assembly for the giant cricket species: *Tarbinskiellus portentosus* (Homchan & Gupta, 2022). However, the utilization of these tools independently resulted in a draft assembly and also needed manual inspection. To improve the assembly process and increase its efficiency, we integrated the Galaxy Europe server as a computational resource. Our current approach integrates automated and manual validation steps to ensure the highest quality of genome assembly.

In the comparative analysis of the PCA plot and the Bayesian Inference‐Based Evolutionary Tree, we observed congruent clustering patterns that provide confidence in the identified groupings. This congruence strengthens the validity of our phylogenetic and genomic analyses. Importantly, both methods highlighted the *A. domesticus* sequences, which are from the same species. These sequences were distinct not only in their position in the PCA plot but also in their placement in the evolutionary tree, suggesting that these particular mitochondrial genomes are divergent from the others in the dataset. This congruence across two different analytical approaches, PCA's dimensionality reduction and Bayesian inference's evolutionary modeling adds robustness to our findings and points to these *A. domesticus* sequences as candidates for further study to understand their unique features or evolutionary trajectories.

In conclusion, this study presents the comprehensive assembly and analysis of the complete mitochondrial genome of the economically important house cricket, *A. domesticus*. The high‐quality mitogenomic data obtained through our robust bioinformatic workflow serve as an invaluable resource for precise DNA barcoding, species identification, and evolutionary studies within the order Orthoptera. The comparative genomic analysis and phylogenetic insights derived from this research shed light on the evolutionary relationships and genetic diversity of *A. domesticus* and related cricket species.

## AUTHOR CONTRIBUTIONS

**Somjit Homchan:** Data curation (equal); funding acquisition (equal); project administration (equal); resources (equal); supervision (equal); writing – review and editing (equal). **Wibhu Kutanan:** Methodology (supporting); validation (supporting); writing – review and editing (supporting). **Yash Munnalal Gupta:** Conceptualization (lead); data curation (equal); investigation (lead); methodology (equal); supervision (lead); writing – original draft (lead).

## CONFLICT OF INTEREST STATEMENT

The authors declare that there are no conflicts of interest.

## Data Availability

The assembled mitochondrial genome sequence which supports this study is available at NCBI (https://www.ncbi.nlm.nih.gov/). Nucleotide sequence data reported are available in DDBJ/ENA/GenBank databases under the accession number OK504623.1. The Assembly workflow can be downloaded for free at https://myexperiment.org/workflows/5163.html.
